# The Value of Ergot in Diseases of the Prostate

**Published:** 1900-07

**Authors:** Eugene R. Corson

**Affiliations:** Savannah, Ga.


### THE VALUE OF ERGOT IN DISEASES OF THE PROSTATE.*

By EUGENE R. CORSON, B.S., M.D.,

SAVANNAH, GA.

For some time I have been using ergot in diseases of the prostate. Reasoning from the standpoint of anatomical homology, it occurred to me that ergot, which has a specific action on the uterus, should also have a specific action on the prostate, for while this gland in its entirety is not perfectly the homologue of the uterus, it contains the *sinus pocularis* or *vesicula prostatica*, discovered by Weber, who, recognizing its homology, called it the *uterus masculinus*. Its homology is based upon the fact that it is developed from the united ends of the rudimentary mullerian ducts. I have failed to find in the literature any reference to this special use of ergot based on this reasoning.

I first began using this drug in congestion and inflammation of the prostatic urethra resulting from gonorrhea. It is in this condition of all others affecting the prostate that ergot proves so useful. How often we are called upon to prescribe for the well-known symptoms of this trouble—sharp pains and burning at the base of the bladder, with strangury, and the passage of blood. These symptoms are apt to come on after a sudden stoppage of the discharge. Examination of the prostate per rectum will show the seat of the trouble. Ergot in these cases will act well alone or in conjunction with gonorrheal remedies. Where pain is the chief symptom, and this may prove to be very distressing, I combine

* Paper read before the Georgia Medical Society, May 8, 1900.

ergot with a mixture I am in the habit of giving with benefit in after-pains, thus still further carrying out the analogy. It is the following:

℞ Tr. aconiti........................................ʒ i.

Tr. gelsemii........................................ʒ ii.

Antipyrin........................................ʒ ii.

Ergotol........................................℥ i.

Aquam ad........................................℥ vi.

M. Sig.: Dessertspoonful every 1, 2 or 3 hours in wineglassful of water.

The ergotol in this prescription may be given separately in alternation with the fever and anodyne mixture. When given alone I prescribe larger doses, as much as a drachm every two or three hours, until relieved, or until a sufficient quantity of the drug has been taken to test its action.

Again, reasoning from the standpoint of homology I often combine ergot with a potash salt. Long ago Lawson Tait pointed out that potash salts reduced the circulation in the uterus and controlled uterine hemorrhage. It seemed quite immaterial which potash salt was used, the bromide, the chlorate, the acetate, or even the iodine, controlling the hemorrhage. However, he used mostly the bromide and the chlorate, and these two drugs I have also used with good results. It is of course necessary to differentiate your case and use that preparation which seems especially indicated. If pain is the predominant symptom the bromide is to be thought of; if a diuretic effect is wanted, the acetate would suggest itself; if the mucous membranes seem chiefly at fault the chlorate will be the drug. My own experience leads me to regard this last potash salt as the most efficient in uterine hemorrhage, though when dealing with the prostate I usually prescribe the bromide. I have had such good results with this drug in combination with ergot in prostatic irritation that I feel that I can recommend it to the profession with much confidence.

There is another condition associated with prostatic trouble, be it limited to the urethral portion, or when the body of the gland is affected, when ergot pure and simple will help us out; I refer to certain forms of sexual weakness dependent upon some prostatic inflammation. A young man comes to us complaining that while the sexual desire is strong, the sexual act is very imperfectly performed, or almost impossible, on account of a premature orgasm and seminal discharge. A careful examination of these cases will show that the sexual center in the cord is not affected, but that the fault lies in the nerve mechanism of the ejaculatory ducts, a hypersensitiveness due to some prostatic irritation. Prostatic mucus can readily be "milked out" by a massage of the prostate per rectum. While these cases are much benefited and cured by sounds and the local application of nitrate of silver, ergot will prove a most efficient drug in conjunction with these remedies, and even alone, for I have relieved a number of such cases in this way. I have cured cases which have been without benefit under a treatment directed to the spinal center in the cord, a part evidently not affected. This form of weakness occurs in young men when the sexual center is probably over-active; the prostate is the seat of the trouble. Of course the bromide should not be used in this condition, as it puts to sleep the spinal center. If the prostate alone is affected give ergot only; if there is any cystitis a bladder remedy can be used to advantage in addition, such as buchu, pareira brava, salol, or the essential oils.

Again there is a condition which is almost the antithesis of this. The sexual function is strong and the patient has been over-indulging and complains of backache, pain at the base of the bladder, and a discharge of prostatic and urethral mucus which he takes to be seminal fluid. Here again the ergot will help us out, and the bromide will now come in as a useful adjuvant, as it quiets the spinal center and takes the place of the will-power, often so powerless when this particular function is concerned.

In the chronic forms of prostatic trouble, the prostatic enlargement of later life, ergot will again help us, though its curative power here must be limited, and any brilliant result out of the question. I should, however, always advise its use in conjunction with the proper surgical treatment. While from the scientific standpoint the one drug seems to be the ideal treatment, from a practical standpoint we must write our prescriptions on an architectonic principle; our prescription must be built up, the keystone must have its other supports to strengthen it and to hold it. This principle can be carried out here with good effect, and our main drug made doubly efficient by adding to it those remedies which seem indicated in the varying conditions of age, temperament, constitution, and the many factors which go to make each case an individual one, a law unto itself.

My object in this short paper is to call the attention of the profession to ergot as a valuable drug when the prostate is involved. I am sure its use in other hands will be followed by equal success.

